# Potential exposure to Australian bat lyssavirus, Queensland, 1996-1999.

**DOI:** 10.3201/eid0603.000305

**Published:** 2000

**Authors:** B J McCall, J H Epstein, A S Neill, K Heel, H Field, J Barrett, G A Smith, L A Selvey, B Rodwell, R Lunt

**Affiliations:** Brisbane Southside Public Health Unit, Cooper's Plains, Queensland, Australia. mccallb@health.qld.gov.au

## Abstract

Two human deaths caused by Australian bat lyssavirus (ABL) infection have been reported since 1996. Information was obtained from 205 persons (mostly adults from south Brisbane and the South Coast of Queensland), who reported potential ABL exposure to the Brisbane Southside Public Health Unit from November 1,1996, to January 31, 1999. Volunteer animal handlers accounted for 39% of potential exposures, their family members for 12%, professional animal handlers for 14%, community members who intentionally handled bats for 31%, and community members with contacts initiated by bats for 4%. The prevalence of Lyssavirus detected by fluorescent antibody test in 366 sick, injured, or orphaned bats from the area was 6%. Sequelae of exposure, including the requirement for expensive postexposure prophylaxis, may be reduced by educating bat handlers and the public of the risks involved in handling Australian bats.


### Potential Exposure to Australian Bat Lyssavirus, Queensland,1996–1999

**Bradley J. McCall,* Jonathan H. Epstein,* Annette S. Neill,* Karen Heel,* Hume Field,† Janine Barrett,‡ Greg A. Smith,§ Linda A. Selvey,¶ Barry Rodwell,† and Ross Lunt#**
*Brisbane Southside Public Health Unit, Cooper's Plains, Queensland, Australia; †Queensland Department of Primary Industries, Yerongpillly, Queensland, Australia; ‡The University of Queensland, St. Lucia, Australia; §Queensland Health Scientific Services, Cooper's Plains, Queensland, Australia; ¶Queensland Health; and #CSIRO Australian Animal Health Laboratory, Geelong, Victoria, Australia

Two human deaths caused by Australian bat lyssavirus (ABL) infection have been reported since 1996. Information was obtained from 205 persons (mostly adults from south Brisbane and the South Coast of Queensland), who reported potential ABL exposure to the Brisbane Southside Public Health Unit from November 1,1996, to January 31, 1999. Volunteer animal handlers accounted for 39% of potential exposures, their family members for 12%, professional animal handlers for 14%, community members who intentionally handled bats for 31%, and community members with contacts initiated by bats for 4%. The prevalence of Lyssavirus detected by fluorescent antibody test in 366 sick, injured, or orphaned bats from the area was 6%. Sequelae of exposure, including the requirement for expensive postexposure prophylaxis, may be reduced by educating bat handlers and the public of the risks involved in handling Australian bats.

Australian bat lyssavirus (ABL) was first reported in July 1996 in a black flying fox (***Pteropus alecto***) from Ballina, New South Wales (1,2). ABL has been confirmed in five species of Australian bat: four species of flying fox (suborder Megachiroptera, genus Pteropus) and one species of insectivorous bat (suborder Microchiroptera, Saccolaimus flaviventris). Two cases of human ABL infection have been reported. The first case occurred in a 39-year-old female animal handler from Rockhampton, Queensland, in November 1996, within 5 weeks of her being scratched and possibly bitten by a yellow-bellied sheath-tailed bat (***S. flaviventris***) (R. Taylor, pers. comm.). The second case occurred in a 27-year-old woman from Mackay, Queensland, in December 1998, >2 years after a bite from a flying fox. Both patients died (3,4).

Address for correspondence: Bradley J. McCall, Brisbane Southside Public Health Unit, P.O. Box 333, 39 Kessels Road, Cooper's Plains, Qld 4108, Australia; fax: 61-7-3000-9130; e-mail: mccallb@health.qld.gov.au.

ABL is a member of the family Rhabdoviridae. Although ABL possesses marked serotypic, antigenic, and molecular sequence similarities to classic rabies virus, it represents a distinct, new genotype, genotype 7 of the Lyssavirus genus (5). The clinical signs of ABL infection in the two human cases were consistent with those of classic rabies infection and included a diffuse, nonsuppurative encephalitis that led to death (3,4). Bats with ABL infection are frequently reported to have had hind limb paresis. While most infected bats are depressed when found, some exhibit uncharacteristic aggression toward humans or other bats. Frequently, a nonspecific, nonsuppurative meningoencephalitis is seen in brains of infected animals (6,7). Vaccine protection trials in mice conducted at the Centers for Disease Control and Prevention (CDC), Atlanta, Georgia, supported the decision to use human diploid cell vaccine (HDCV) for human ABL prophylaxis (7-9). Historically, Australia has been considered free of rabies and rabieslike viruses. Thus, before the first human case of ABL infection in 1996, no measures existed to prevent rabies or rabieslike disease acquired as a result of contact with Australian domestic animals or wildlife. Since the first human ABL case, the Queensland Health Department, in accordance with the recommendations of the national Lyssavirus Expert Group, has provided postexposure prophylaxis (PEP) to persons who report potential exposure to ABL through bites, scratches, and permucosal or percutaneous exposure to bat saliva or neural tissue (9,10). Preexposure prophylaxis is recommended for persons who report frequent contact with bats.

Colonies of flying foxes are common in suburban areas of southeast Queensland. The black flying fox (***Pteropus alecto***) and the grey-headed flying fox (***P. poliocephalus***) live there throughout the year, and the little red flying fox (***P. scapulatus***) occurs seasonally. While the population of flying foxes may be decreasing in southeast Queensland, fragmentation of colonies has resulted in a wider distribution of smaller colonies (L. Hall, pers. comm.). Direct contact with bats by the general public and animal handlers is not uncommon (11). Volunteer animal handlers rehabilitate sick, injured, and orphaned bats and are frequently bitten, scratched, or exposed to bat saliva. Since November 1996, the Brisbane Southside Public Health Unit (BSPHU) and other state public health units have been involved in coordinating lyssavirus PEP. This article describes the pattern of potential human exposure to ABL reported to the Communicable Disease Control Section of BSPHU between November 1996 and January 1999 and subsequent PEP. Disease prevalence findings are presented for bats surveyed in southeast Queensland by the Animal and Plant Health Service of the Queensland Department of Primary Industries.

### The Study

During the study (November 1, 1996, to January 31, 1999), the Communicable Disease Control Section of BSPHU served a population of approximately 1.1 million persons in several local government areas, south Brisbane (part of the Brisbane City Council Area), Logan, Redlands, Beaudesert, and Gold Coast (12). All persons who reported a potential ABL exposure (bat bite, scratch, percutaneous, or permucosal exposure to bat saliva or neural tissue) were asked to complete a standard questionnaire, which sought demographic information (including occupation, history of professional or volunteer bat handling, history of rabies vaccination, potential rabies exposure [bite, scratch, provoked, unprovoked], circumstances that led to the exposure, treatment received, and any laboratory investigation of the bat).

A separate questionnaire was completed for each occasion a person contacted BSPHU to report potential ABL exposure. Potential exposures were reported retrospectively, and the dates of notification and potential exposure for each case were included. All information was recorded and analyzed by using an Epi-Info 6.04b database (13). Age and gender-specific notification rates were calculated by using estimated resident population data for 1997 (12).

During the same period, healthy bats, sick and injured bats, and bats involved in a potential human exposure to ABL were tested for infection with a fluorescein-labeled antirabies monoclonal globulin (CENTOCOR) in a direct fluorescent antibody test (DFAT) on fresh brain impression smears at the Queensland Department of Primary Industries Animal Research Institute or at the CSIRO Australian Animal Health Laboratories. Material from most bats that tested positive for ABL infection and from bats associated with a potential human exposure to ABL were sent to either the Australian Animal Health Laboratory or Queensland Health Scientific Services for confirmation by DFAT, virus isolation, and polymerase chain reaction.

### Results

A total of 205 notifications to BSPHU met the criteria for potential ABL exposure during the study period, an average annual notification rate of 8.1/100,000. Complete information was obtained from 202 persons. Total notifications included 86 males and 119 females (M:F ratio of 1:1.38). The age- and gender-specific average annual notification rates are presented in Figure 1. Most reported potential exposures (116 of 204) were among persons 19 to 49 years of age. The months of potential ABL exposure and notification are presented in Figure 2. Most notifications (131 of 205) were made within 2 months of each of the two fatal human cases. Nine (11%) of 80 notifications made in the 2 months following the first reported case were related to exposures that occurred >2 months before the first human case was publicized. The median interval between

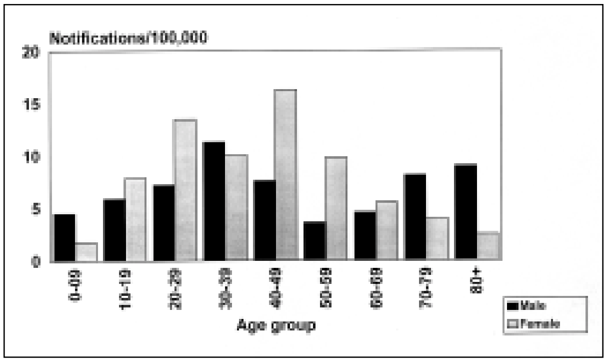

Figure 1. Age and gender-specific average annual notification rates of potential human exposure to Australian bat lyssavirus (n = 204) south Brisbane and South Coast, Queensland, 1996-1999.

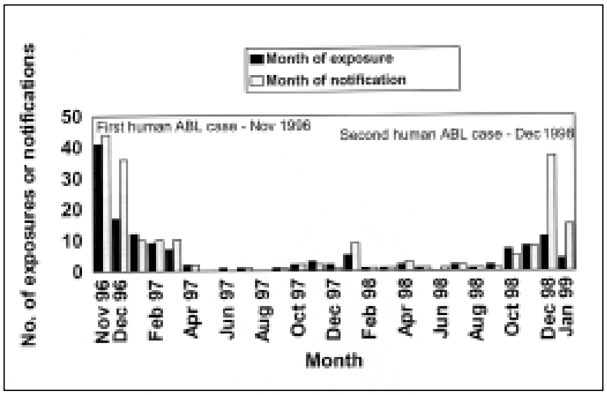

Figure 2. Dates of potential Australian bat lyssavirus exposures and notifications to the Brisbane Southside Public Health Unit, south Brisbane and South Coast, Queensland, 1996-1999.

exposure and notification of these 80 potential exposures was 17 days (0 to 1,080 days). In the 2 months following the second case, 22 (43%) of 51 notifications were related to potential exposures that had occurred before the reporting of the first human case. The median interval between exposure and notification of the 51 potential exposures was 728 days (0 to 2,907 days). A further 14 (27%) of the 51 notifications were related to potential exposures that had occurred since the first human case but had not been reported to BSPHU at the time of exposure.

### Season of Exposures

Potential exposures to ABL were reported to have occurred from 1991 to 1999, most during spring and summer (September to February) (n = 151, 74%). While the occurrence of the two human ABL cases in spring and summer influenced the reporting of potential exposures at these times, this trend of increased spring-summer potential exposures persisted in the period between the two reported human ABL cases. The highest number of potential exposures (105) was reported in the year of the first human case; 99 occurred in the spring or summer of 1996-97.

### Groups at Risk

Notifications were categorized according to the person's life-style and occupational potential for exposure to ABL (Table). The group at highest risk, volunteer bat handlers, reported 79 (39%) potential exposures; 8 of these handlers reported a second potential exposure during the study.

Table. Groups at risk for exposure to Australian bat lyssavirus

| Groups at risk | No. of potential exposures (n=203) | Mean age and age range (yrs) | Gender (m/f) | Median interval between exposure and notification (d) | Bite/nonbite injury (n=202) | Provoked (%) (n=202) |
|---|---|---|---|---|---|---|
| Volunteer bat handlers | 79 | 40.5 (16-83) | 15/64 | 19 (0-2,105) | 56/23 | 79/79 (100) |
| Household or family member of volunteer bat handlers | 24 | 17.5 ( 5-51) | 12/12 | 27 (0-1,809) | 18/6 | 24/24 (100) |
| Professional animal worker | 28 | 34 (17-69) | 15/13 | 4 (0-1,818) | 13/14 | 27/27 (100) |
| Community-intentional potential exposure | 63 | 39 ( 6-85) | 40/23 | 10 (0-2,907) | 41/22 | 62/63 ( 98) |
| Community-unintential exposure | 9 | 32 (16-49) | 4/5 | 2 (0-32) | 3/6 | 4/9 ( 44) |

Twenty-four (12%) notifications of potential exposure were among household or family members of volunteer bat handlers. Professional animal handlers (e.g., veterinarians, wildlife biologists, park rangers) reported 28 (14%) exposures. Community members who handled bats (usually in the course of freeing them from a fence or entanglement) reported 63 (31%) potential exposures. Community members reported 9 unintentional potential exposures in which contact was initiated by the bat.

The pattern of notification varied within groups at risk during the study. The number of potential exposures reported by volunteer and professional animal handlers declined. Notifications by all groups were highest in the months after the reported fatal human cases of ABL infection. The number of potential exposures reported in the 2 months after the first human case (n = 80) was higher than after the second case (n = 51), particularly among volunteer bat handlers, who reported the highest number of potential ABL exposures in the 2 months after the first human case (43 [53%] of 80), decreasing to 12 (24%) of 51 in the 2 months after the second case. Notifications of potential exposures among community members who had intentionally handled bats rose from 11 (14%) of 80 in the 2 months after the first human ABL case to 23 (45%) of 51 in the 2 months after the second human ABL case.

### Nature of Exposure

Potential exposures were classified as bite or nonbite exposures in accordance with international recommendations (14). Most potential exposures were bites (n = 132, 64%). The ratio of bite to nonbite potential exposures within groups at risk was highest among volunteer bat handlers (56:23) (Table). Potential exposures associated with unintentional contact with bats by community members were predominantly scratches (3 bite: 6 nonbite), whereas potential exposures from intentional contact with bats by all other risk groups were predominantly by bites (128 bites: 65 nonbites). Potential exposures were categorized as provoked (arising from intentional contact with a bat) or unprovoked (a contact initiated by the bat). Most potential exposures (97%) were described as provoked (Table).

### Treatment

PEP was offered to all persons who reported potential ABL exposures, in accordance with international and Australian recommendations (8,14). Standard PEP for unvaccinated persons consisted of human rabies immune globulin (HRIG, 20 IU/kg) on day 0 and 5 doses of HDCV administered on days 0, 3, 7, 14, and 28. PEP for immunized persons consisted of 2 booster doses of HDCV administered on days 0 and 3. A national shortage of HRIG required modifications to the standard PEP regimen. Sixty-two potentially exposed persons received standard PEP, 100 received 5 doses of HDCV only, 16 vaccinated persons received 2 booster doses of HDCV, and 25 persons did not receive treatment when the bat tested negative. Two persons refused vaccination because of concerns about potential vaccine side effects. Sixteen persons ceased treatment when the bat tested negative.

The estimated cost of providing PEP during this study was A$137,368, which included A$30,930 for medical services funded through the Commonwealth Medicare system (calculated on the cost of six visits to a medical practitioner for each person requiring a 5-dose course of PEP and three visits for each person requiring 2 doses of PEP); A$8,200 for public health officers who interviewed potentially exposed persons; A$10,600 for laboratory testing of the bats; and A$87,638 for HDCV and HRIG. The cost of all vaccines was met by the Queensland Health Department.

### ABL Test Results in Bats

All bats retrieved from a human exposure incident underwent postmortem examination and testing for ABL infection. Thirty-six bats were tested; two were positive on DFAT and polymerase chain reaction testing for Lyssavirus—a black flying fox and a little red flying fox. The tested bats included 13 black flying foxes, 11 grey-headed flying foxes, 5 little red flying foxes, and 7 insectivorous bats.

In a separate investigation, the Queensland Animal Research Institute tested bats by DFAT on brain impression smears for evidence of ABL infection since June 1996. From November 1, 1996, to January 31, 1999, some 153 healthy wild-caught flying foxes; 181 healthy wild-caught insectivorous bats; 366 sick, injured, or orphaned flying foxes; and 39 sick or injured insectivorous bats from the area served by BSPHU and greater Brisbane were tested. Of these, 21 (6%) of the 366 sick, injured, or orphaned flying foxes tested positive for ABL infection, including the 2 involved in human exposures in the BSPHU area. All other bats tested negative.

### Discussion

This is the first description of PEP provided to an Australian community after the recognition of human risk for ABL infection. The first human case triggered a large national public health campaign and considerable public awareness about the risks from bats, particularly in communities such as south Brisbane and the South Coast of Queensland, where large colonies of bats live close to human urban populations and bat/human interaction is not uncommon. Increased concern was demonstrated by the large number of notifications of potential exposure that followed reports of the two human cases (Figure 2). Most potential human exposures were among adults (ages 25 to 49). The increased proportion of women reflects the high proportion of female volunteer bat handlers in the study population.

The first 2 months of notifications represents a catch-up period in which PEP was provided to persons with exposures dating back several years. Relatively few notifications occurred after the first 2 months of the study, and it was only after the second human case, which had an assumed incubation period of approximately 2 years, that another cluster of notifications occurred (4). The median interval between potential exposure and notification increased from 17 days for those notified in the 2 months after the first human ABL case to 728 days for those notified in the 2 months after the second case. The potential exposures reported in the 2 months after the second case included 22 persons who were potentially exposed before the first case and 14 with >1 month between potential exposure and notification. The second human case with its prolonged incubation period reinforced the public perception of the severity of this disease and prompted more notifications.

Potential exposures occurred most commonly in spring and summer, coinciding with the birthing season (October to December) of the black and grey-headed flying foxes in southeastern Queensland (15). During each birthing season in southeastern Queensland, 100 to 300 neonatal and juvenile black or grey-headed flying foxes are reared by volunteer bat handlers (H. Luckhoff, pers. comm.). These orphans are commonly assumed to have been abandoned or separated from their dams. Frequently, orphans are found still clinging to the body of their dam. Further research is required to identify any association between orphaned bats and the ABL status of the dam. A case of clinical disease in an in-care juvenile black flying fox and the associated exposure of eight humans has been described (6).

Most potential exposures (107 [52%] of 205) were reported from groups who handled bats. These groups were the target of initial public health information campaigns to raise awareness of the risks for ABL infection. PEP was provided to members of these groups after the first human case, and a recommendation was issued that all workers in these fields be vaccinated with HDCV and that unvaccinated persons, including family members of volunteer bat handlers, not handle bats. Seventy-two (35%) of 205 potential exposures occurred among members of the community. Most of these (63 [88%] of 72) had rescued a trapped or fallen bat. The test results from bats indicate that sick, injured, or orphaned bats have a significantly higher crude prevalence of ABL infection ($p < 0.001$) than healthy wild-caught or captive bats. Consequently, the risk for ABL exposure among volunteer and professional bat handlers and persons who rescue bats may be relatively increased because these groups primarily handle sick, injured, or orphaned bats.

Reporting of potential exposures among groups at risk changed with time during the study. One important factor in the management of PEP was the requirement (introduced in 1997) that all bats involved in a potential human exposure be surrendered for postmortem examination and laboratory testing for ABL. Those who care for bats are often reluctant to surrender them for ABL testing. Notifications from volunteer bat handlers declined during the study period. While this may reflect a decline either in the number of bat handlers or in potential exposures among volunteer bat handlers, underreporting may be occurring in this group. Anecdotal evidence suggests that this reduction in notifications may reflect handlers' concern for the bats. Such underreporting could be associated with future human cases. Most potential exposures resulted from intentional handling of bats. The few potential exposures from unprovoked encounters suggest that bats rarely initiate contact with humans.

The recognition of ABL infection has resulted in a large public health program to provide education, counseling, and prophylaxis to volunteer and professional bat handlers and members of the community who may be exposed to ABL. The focus of the program has been to encourage preexposure vaccination of bat handlers, prevention of potential exposures by avoidance of bat handling by nonvaccinated persons, and prompt medical care when potential exposures occur. The cost of PEP for all those potentially exposed to ABL in south Brisbane and the South Coast of Queensland during the study was considerable. Future public health interventions should continue to emphasize the risks associated with interaction with bats to reduce the requirement for PEP and the likelihood of human cases of ABL infection.

### Acknowledgments

The authors thank Russell Stafford, Helen Luckhoff, Les Hall, Nicki Markus, Bruce Harrower, David Gould, the Queensland Medical Laboratory, and the medical practitioners of south Brisbane and South Coast for their assistance in aspects of patient management, laboratory investigation, vaccine delivery, and for advice throughout the study.

Mr. Epstein's work was supported by grants from the National Institutes of Health, the Hickey-Peyton International Travel Fellowship, and the Department of International Programs, Tufts University School of Veterinary Medicine.

Dr. McCall, a public health physician, has led the Communicable Disease Control team at the Brisbane Southside Public Health Unit for 5 years. His research interests include meningococcal disease, leptospirosis, and ABL.

### References

1. Crerar S, Longbottom H, Rooney J, Thornber P. Human health aspects of a possible lyssavirus in a flying fox. Commun Dis Intell 1996;20:325.
2. Fraser G, Hooper P, Lunt R, Gould AR, Gleeson LJ, Hyatt AD, et al. Encephalitis caused by a lyssavirus in fruit bats in Australia. Emerg Infect Dis 1996;2:327-31.
3. Allworth A, Murray K, Morgan J. A case of encephalitis due to a lyssavirus recently identified in fruit bats. Commun Dis Intell 1996;20:504.
4. Mackenzie J. Emerging viral diseases: an Australian perspective. Emerg Infect Dis 1999;5:1-8.
5. Gould A, Hyatt A, Lunt R, Kattenbelt JA, Hengstberger S. Characterisation of a novel lyssavirus isolated from Pteropid bats in Australia. Virus Res 1998;54:165-87.
6. Field H, McCall B, Barrett J. Australian bat lyssavirus infection in a captive juvenile black flying fox. Emerg Infect Dis 1999;5:438-40.7.
7. Hooper P, Lunt R, Gould A, Samaratunga H, Hyatt AD, Gleeson LF, et al. A new lyssavirus—the first endemic rabies-related virus recognized in Australia. Bulletin Institut Pasteur 1997;95:209-18.
8. Rabies and bat lyssavirus infection. In: Watson C, editor. The Australian immunisation handbook. 6th ed. Canberra: Australian Government Publishing Service, 1997:162-8.
9. Lyssavirus Expert Group. Prevention of human lyssavirus infection. Commun Dis Intell 1996;20:505-7.
10. Lyssavirus Expert Group. Update on bat Lyssavirus. Commun Dis Intell 1996;20:535.
11. Birt P, Markus N, Collins L, Hall L. Urban flying foxes. Nature Australia 1998;Spring:55-9.
12. Australian Bureau of Statistics. 1997 estimated resident population by statistical local area. Australian Bureau of Statistics catalogue no. 3235.3. Canberra, Australia: The Organization,1997.
13. Dean AG, Dean JA, Coulombier D, Brendel KA, Smith DC, Burton AH, et al. Epi-Info, version 6.04b: a word processing, database, and statistics system for epidemiology on microcomputers (computer program). Atlanta, GA: Centers for Disease Control and Prevention, 1997.
14. Centers for Disease Control and Prevention. Rabies Prevention—United States, 1991. Recommendations of the Immunization Practices Advisory Committee. MMWR Morb Mortal Wkly Rep 1991;40:R3:1-19.
15. Hall LS. Black flying fox. In: Strachan R, editor. The mammals of Australia. Chatswood, Australia: Reed Books 1995;432-7.
